# Comparison of the Efficacy and Safety of Atazanavir/Ritonavir Plus Hydroxychloroquine with Lopinavir/Ritonavir Plus Hydroxychloroquine in Patients with Moderate COVID-19, A Randomized, Double-blind Clinical Trial

**DOI:** 10.22037/ijpr.2021.115157.15243

**Published:** 2021

**Authors:** Zahra Nekoukar, Shahram Ala, Siavash Moradi, Andrew Hill, Ali Reza Davoudi Badabi, Ahmad Alikhani, Shahriar Alian, Minoo Moghimi, Amir Mohammad Shabani, Hamideh Abbaspour Kasgari

**Affiliations:** a *Department of Clinical Pharmacy, School of Pharmacy, Mazandaran University of Medical Sciences, Sari, Iran. *; b *Education Development Center, Mazandaran University of Medical Sciences, Sari, Iran. *; c *Department of Translational Medicine, University of Liverpool, UK. *; d *Antimicrobial Resistance Research Center, Department of Infectious Diseases, Mazandaran University of Medical Sciences, Sari, Iran.*

**Keywords:** COVID-19, Atazanavir, Lopinavir, Hospital stay, Mortality, Safety

## Abstract

This was a randomized, double-blind clinical trial to compare the efficacy and safety of Atazanavir/Ritonavir (ATZ/RTV) with Lopinavir/Ritonavir (LPV/RTV) in moderate Coronavirus disease 2019 (COVID-19). Participants were randomly assigned to receive a single dose of hydroxychloroquine (HCQ) plus ATZ/RTV or LPV/RTV for a minimum of 5 to a maximum of 10 days. The primary outcomes were the reduced length of hospital stay and clinical recovery within 10 days from starting the intervention. The rate of intensive care unit (ICU) admission, intubation, and mortality, the lengths of ICU stay and being intubated, recovery within 14 days, and the frequency of adverse reactions were considered as secondary outcomes. Among 132 enrolled patients, 62 cases in each arm were analyzed at the end of the intervention. Fifty-one (82.3%) cases in the ATZ/RTV arm versus 41 (66.1%) in the LPV/RTV arm were discharged within 10 days (*P = *0.06). The median number of the intervention days was 6 (IQR: 5-8) in ATZ/RTV arm versus 7 (IQR: 6-9) in LPV/RTV arm (*P = *0.01). The rate and length of ICU admission and intubation (*P *≥ 0.99), rate of mortality (*P = *0.49), and recovery within 14 days (*P = *0.09) were not statistically different between groups. The most reported adverse reactions were nausea and vomiting that all cases were in the LPV/RTV arm (*P* = 0.006). ATZ/RTV is better tolerated in comparison with LPV/RTV; however, it did not show more efficacy than LPV/RTV in clinical outcomes of COVID-19 in this study.

## Introduction

Coronaviruses (CoVs) belong to the family of *Coronaviridae* with a single-stranded positive-sense enveloped RNA (1). In late December 2019, Severe acute respiratory syndrome Coronavirus 2 (SARS-CoV-2) emerged in Wuhan, Hubei Province, China, and rapidly spread throughout the world and was announced as a pandemic by the World Health Organization (WHO) on 11th March 2020 (2). Many intensive efforts are ongoing worldwide to produce new drugs and SARS-CoV-2 vaccines, but since their evolution is a time-consuming process, no definitive drug has been yet discovered to treat infected patients, and investigations are focused on existing investigations drugs. Numerous available antiviral agents have been evaluated in sequence analysis, modeling, and docking studies, and some of them exert potential efficacy to inhibit different molecular targets in SARS-CoV-2 (3, 4). Remdesivir, Favipiravir, Arbidol (Umifenovir), Interferon, Sofosbuvir/Daclatasvir, Ribavirin, and Lopinavir/ritonavir (LPV/RTV) are the antiviral agents that have been evaluated in several clinical trials for Coronavirus disease-2019 (COVID-19) (5-11). LPV/RTV is active against human immunodeficiency virus (HIV) and previously showed efficacy against the two highly infectious types of CoVs, severe acute respiratory syndrome (SARS) and Middle East respiratory syndrome coronavirus (MERS) (12, 13). H. Zhong, *et al.* reported that the combination regimens with LPV/RTV result in better viral eradication and improved radiographic findings with fewer acute respiratory distress syndrome (14). Atazanavir (ATZ) (Reyataz^®^), as an azapeptide oral protease inhibitor (PI), was approved by the food and drug administration (FDA) in 2003 for HIV infection (15). ATZ is generally well-tolerated, and possible common side effects include headache, nausea, abdominal pain, and diarrhea. An increase in unconjugated bilirubin may occur and is often benign and reversible after discontinuation of the drug (16). With concomitant administration of RTV 100 mg/day, ATZ AUC_0-24 _and minimum plasma concentration were notably increased (17, 18). The drug is also a moderate inhibitor of cytochrome P450 (CYP) 3A4 leading to potential drug-drug interactions with other CYP3A4 substrates (19). Drug target interaction studies showed inhibitory effects of ATZ on the main protease (pro, also called 3CLpro) of SARS-CoV-2 (20, 21). It was also proven in another research by Density Functional Theory method that there are 4 interactive positions in SARS-CoV-2 which interact with the molecular structure of ATZ through hydrogen bonds resulting in protein degradation of the virus (22).

Considering potential benefits of ATZ to inhibit virus replication from molecular studies, and also a recent study suggesting ATZ efficacy in reducing the duration of hospital stay (23), this randomized, double-blind clinical trial designed to compare the efficacy and safety of ATZ/RTV with LPV/RTV in moderate COVID-19.

## Experimental

*Ethics and registration number*

This study was in accordance with the National Institutes of Health Guidelines (NIH). The Ethics Committee of Mazandaran University of Medical Sciences approved the study protocol (IR.MAZUMS..REC.1399.8043). The study protocol was also registered in the Iranian Registry of Clinical Trials (ID Code: IRCT20200328046886N2), accessible at https://www.irct.ir/trial/50980.

*Design and Participants*

This randomized, double-blind clinical trial was performed from 22 August 2020 to 21 November 2020 in Razi Hospital, the epicenter of the COVID-19 outbreak located in Qaemshahr City, Mazandaran Province, Iran. All patients with a positive nasopharyngeal swab reverse transcription-polymerase chain reaction (RT–PCR) or those with COVID-19 compatible spiral lung Computed Tomography (CT) Scan were assessed for eligibility. Inclusion criteria were 1) Informed consent form 2) age 18-75 years, 3) Onset of symptoms ≤7 days, 4) COVID-19 symptoms (Fever (oral temperature ≥37.8 °C at any one time before enrolment), Dry cough, Severe fatigue, or Dyspnea), 5) Lung CT compatible with moderate COVID-19 (Involvement of up to 3 or 4 lung lobes with an area less than one-third of the volume of each lobe or involvement of one or two lobes with a larger area in the CT scan of the patient), 6) Arterial O_2_ saturation (O_2_ Sat%) 90-93%. The patient must pass the items 1 to 4 plus one of the items 5 or 6.

Exclusion criteria were: 1) Hepatic Failure (Child-pugh C), 2) Taking carbamazepine/rifampin/phenytoin, 3) Previous confirmed COVID-19 infection, 4) Enrollment in other trials, 5) Pregnancy/lactation, 6) Immune suppression/compromised, 7) Active cancer, 8) Multi-organ failure (≥2 organs), 9) Requiring intubation on admission, 10) Significant arrhythmia in electrocardiography, 11) A known allergic reaction to ATZ, LPV or RTV, and 12) Severe disability preventing cooperation.

*Randomization and Blinding*

One hundred and thirty-two patients with confirmed COVID-19 who had met the inclusion criteria and signed an informed consent form were randomly assigned in two intervention arms using Sealed envelope online software to make block size 4. The investigator and data analyzer were blind to the type of intervention, while the patient and physician were not.

*Procedures*

In one group, patients received 400 mg single dose of hydroxychloroquine (HCQ) (Modaquenil^®^, Mofid Pharmaceutical co., Iran) plus one tablet of Atazanavir sulfate/Ritonavir 300/100 mg (ANZAVIR-R^®^, Mylan Laboratories Limited, India) for a minimum of 5 to a maximum of 10 days, whereas in other group patients received 400 mg single dose of HCQ plus 2 tablets of Lopinavir/Ritonavir 200/50 mg (RITOVIR^®^, Nordic Pharmaceutical co., Sweden) twice daily for a minimum of 5 to a maximum of 10 days. Other COVID-19 related drugs that patients consumed during the intervention such as antivirals, Corticosteroids, non-steroidal anti-inflammatory drugs (NSAIDs), Antibiotics, Anticoagulants, Intravenous immunoglobulin (IVIG), and Tocilizumab, were recorded.

*Data collection*

At baseline, demographic data, underlying diseases include Hypertension (HTN), Diabetes Mellitus (DM), Ischemic Heart Disease (IHD), Asthma, Chronic Obstructive Pulmonary Disease (COPD), Hypothyroidism, and Gastrointestinal (GI) symptoms include abdominal pain/discomfort, diarrhea, nausea and vomiting, and loss of appetite were collected. Physical examination, including O^2^ Sat%, pulse rate, respiratory rate (RR), blood pressure, temperature, body mass index (BMI), PCR result if done, and percentage of lung involvement, was also recorded at the time of hospital admission. Baseline laboratory tests such as complete blood cell count (hemoglobin, platelet, white blood cells (WBC), polymorphonuclear leukocytes (PMN) and lymphocytes (Lymph), serum electrolytes (sodium and potassium), blood sugar, liver enzymes, total and direct bilirubin, blood urea nitrogen, international normalized ratio, inflammatory biomarkers consisting C-Reactive Protein (CRP) and erythrocyte sedimentation rate, vein blood gas pH and bicarbonate were extracted from the patient’s file.

The value of O_2_ Sat% (in room air), RR, and any important events related to either COVID-19 or study medications were evaluated every day during the intervention. On the 3rd and 7th days, CRP, WBC, PMN, Lymph, and total and direct bilirubin were measured. Positive nasopharyngeal swab RT–PCR cases on admission were retested on the 7th day, and if the result were positive, the test would be repeated on the 14th day. The final patients’ outcome during the 10 days of intervention was defined as discharged, death, release sheet, or withdrawal of consent. The release sheet item which was assigned to patients who received at least 5 days of intervention, was considered if the patient experienced any condition related to intervention or COVID-19 that led to discontinuing the intervention. 

*Outcomes*

The primary outcomes of this trial were the reduced duration of intervention and clinical recovery within 10 days of starting the medicine. Clinical recovery was defined as achieving criteria to discharge, including O_2_ Sat% ≥ 95% or ≥5% improvement over baseline in room air, no fever, no dyspnea, improvement/treatment of cough, improvement/treatment of fatigue, and oral tolerance. All these items should be maintained for at least 24 hours. The secondary outcomes included the rate of intensive care unit (ICU) admission, intubation and mortality, the lengths of ICU stay and being intubated, recovery within 14 days from starting the intervention, and the frequency of adverse reactions. Safety outcomes were also measured as frequencies of reported important events. 

*Statistical analysis*

Qualitative variables were reported by frequency and percent, and quantitative variables after determination of the pattern of their distribution by Kolmogorov-Smirnov test were reported by mean/standard deviation or median/interquartile range or minimum-maximum. To compare the differences between the two groups, the Chi-square test (Fisher’s Exact Test), Independent *t*-test, and Mann–Whitney U test was used for qualitative and quantitative variables, respectively. The Intention-to-treat approach was carried out for analysis of the study. Statistical analysis was performed by using SPSS software version 25 (SPSS Inc., Chicago, IL, USA), and values with a *P*-value < 0.05 were considered statistically significant.

## Results

**Figure 1:**
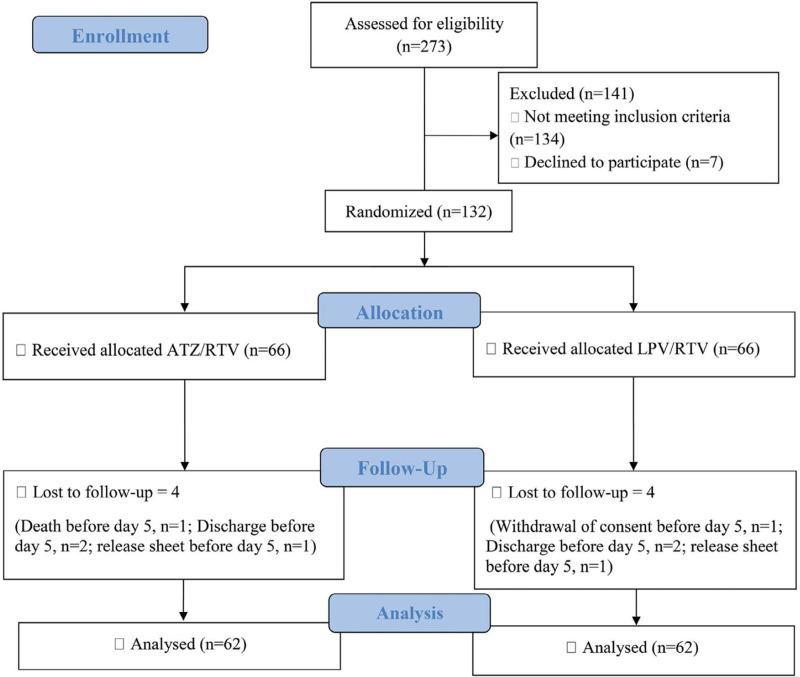
Flow Diagram

Among the patients admitted to the Razi Hospital between 22 August 2020 and 21 November 2020, 273 patients were assessed for eligibility to enter the study. One-hundred and forty-one patients were not eligible, and finally, 132 were enrolled in the study. Sixty-six patients were randomly assigned to each study group to receive either ATZ/RTV or LPV/RTV. One hundred and twenty-four participants passed the minimum 5 days of intervention to be included for the final analysis (62 in each arm) (Figure 1). Sixty-nine (55.6%) patients were male, and the mean age of the participants was 49.95 years ( ± 12.62). There were no statistically significant differences between the two study arms in baseline demographic data, including age, gender, BMI, and underlying diseases, symptoms, physical examination, and finally, laboratory data (Table 1). 

**Figure 2:**
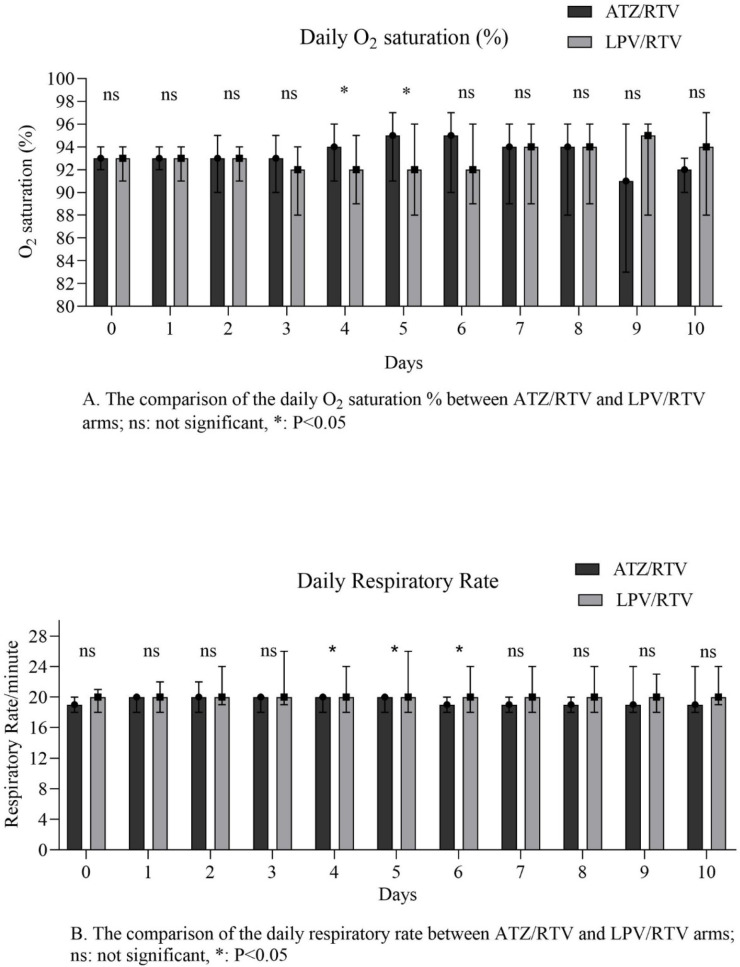
Daily assessment of O_2_ Saturation (%) and Respiratory rate in each group

The results of primary outcomes are shown in table 2. The median number of days taking intervention was significant between the study arms (*P *= 0.01). The number of clinically recovered patients within 10 days from starting the study medication was not statistically significant between the two groups (*P* = 0.06). No significant difference was seen among the secondary outcomes of the study between groups. Results of the measured parameters on days 3 and 7, and also baseline, days 7 and 14 nasopharyngeal swabs RT–PCR were presented in Table 2. In the daily assessment of the O^2^ sat % and RR, the median of the O^2^ sat % on 4^th^, 5^th^, and final days and RR on 4^th^, 5^th^, 6^th^, and final days were different between groups (Figure 2). 

The frequency of the most reported important events by the participants was not significantly different between the two groups except for nausea and vomiting, which were higher in the LPV/RTV arm (8 patients (12.9%) versus none in the ATZ/RTV arm) (*P = *0.006). There was no statistical difference in the case of prescribing other COVID-19-related drugs between the two groups (Table 3).

## Discussion

In this double-blind, randomized clinical trial, the efficacy and safety of ATZ/RTV plus a single dose of HCQ were compared to LPV/RTV plus a single dose of HCQ in moderate COVID-19. More patients were discharged from the hospital within 10 days of intervention in the ATZ/RTV arm; however, it was not statistically significant. Also, ATZ/RTV revealed a more tolerability profile than LPV/RTV, with significantly fewer episodes of nausea and vomiting.

Before designing the present clinical trial, the standard therapeutic regimen used to treat hospitalized moderate COVID-19 patients was LPV/RTV in combination with HCQ in Razi Hospital. According to the use of 2 large size LPV/RTV pills twice a day, possibly with other medications that could alter the patient’s compliance with treatment, the alternative available regimen, ATZ/RTV, was considered. Nausea and vomiting were also the most reported adverse effects with LPV/RTV that reduced the patient’s tolerability. Atazanavir, with a lower pill burden and more tolerable adverse effects rather than other PIs, has been exerted activity against SARS-CoV-2 main protease (M^pro^), in several in vitro experiments (24-26).

The ATZ molecule penetrates well through the respiratory tract and inhibits SARS-CoV-2 replication by interacting with M^pro^ in pulmonary epithelial cell lines. RTV, an inhibitor of CYP 3A4, is responsible for enhancing the inhibitory effect of ATZ on SARS-CoV-2 (27-29). Natalia Fintelman-Rodrigues *et al.* showed that ATZ interaction with M^pro^ enzymatic activity of SARS-CoV-2 is stronger and more stable than LPV (27). They also mentioned that ATZ/RTV prevents cell death and the production of pro-inflammatory cytokines, including interleukin-6 and TNF-alpha in monocytes that SARS-CoV-2 infects. Inhibition of M^pro^, the critical protease of SARS-Cov-2 by ATZ, prevents viral replication leads to blocking the cytokine storm-release associated mediators (30, 31). According to the more discharged cases within 10 days from starting the study medication in ATZ/RTV group, the faster viral clearance with ATZ/RTV with the quoted mechanisms could be declared. It should be mentioned that the median time from symptom onset was 7 days in each group that may be responsible for no significant advantage of ATZ/RTV over LPV/RTV in reducing time to recovery in moderate COVID-19. Although the discharge rate within 10 days was not statistically different among the two arms (P *= *0.06), the clinical recovery trend was in favor of ATZ/RTV (51 *vs.* 41 patients). 

Among the secondary outcomes of the present trial, with the exception of adverse effects, no statistical benefit was obtained by ATZ/RTV. However, two participants who died during the intervention in LPV/RTV versus none in the ATZ/RTV arm are clinically notable. Additionally, the rate and duration of ICU admission and intubation did not reveal any significant differences between the two groups, which may be explained by the study’s limited sample size. It should be considered that this trial was conducted on moderate COVID-19 cases, and this issue can also influence the low rate for ICU admission. However, as concluded by Rahmani H* et al*., ATZ is effective in reducing 28-days and 56-days mortality rates and ICU-admission in moderate COVID-19 (23). Another study by Mazaherpour *et al.* demonstrated no statistical difference between ATZ/RTV and LPV /RTV in improving clinical outcomes, but drug-related side effects differed. Episodes of dysrhythmia more occurred in the LPV /RTV arm, and similar to the present study, hyperbilirubinemia was significant in those treated with ATZ/RTV (32). 

Release sheet was recorded for 19 versus 9 patients in the LPV/RTV and ATZ/RTV arm, respectively, almost promising the superiority of ATZ/RTV. In patients who received LPV/RTV, the most frequently reported causes of release sheet were nausea and vomiting and not reaching expected clinical improvement. Nausea and vomiting obviously reduced patients’ compliance, while; there was no certain adverse reaction interfering with intervention in the ATZ/RTV arm. However, the main cause of the release sheet in the ATZ/RTV group was the failure to achieve the expected clinical improvement. By a closer look at the causes of the release sheet, the number of patients who needed more medical intervention was still higher in the LPV/RTV arm. (Data is available in supplementary material attachment 1) These findings support the potential superiority of ATZ/RTV to ameliorate disease by the less need for administering other antiviral agents. 

Statistically, analysis of each concomitant administered drug for COVID-19 did not show significant differences between the two study arms; therefore, the results could be compared between the two study interventions.

Nausea and vomiting were the most reported adverse reactions in this study that all were in the LPV/RTV arm (8 (12.12%)). Unlike LPV/RTV, ATZ/RTV was well tolerated with an acceptable safety profile. As expected, the values of total and direct bilirubin were significantly higher in the ATZ/RTV group on days 3 and 7 of the study (*P* ≤ 0.001). As seen in our study, ATZ-related hyperbilirubinemia was asymptomatic and transient in most patients (33), and only two patients became icterus which was not statistically different between groups (*P* = 0.49) and resolved after drug cessation. It was interesting that hiccup was an important event that was reported by only 2 patients in ATZ/RTV arm. However, it is not clear whether it was related to the drug or COVID-19. At the time of writing this paper, there was no additional published clinical trial of Atazanavir for COVID-19 to compare the results. Three ongoing clinical trials have been recruited on https://clinicaltrials.gov/ entitled “NA-831, Atazanavir and Dexamethasone Combination Therapy for the Treatment of COVID-19 Infection (NATADEX)”, “Antiviral Agents Against COVID-19 Infection (REVOLUTIOn)”, and “The Nitazoxanide Plus Atazanavir for COVID-19 Study (NACOVID)”. There were also three other recruited trials on https://www.irct.ir/ whose data has not been released yet. 

To compare the efficacy of ATZ/RTV versus LPV/RTV against COVID-19, this study faced some limitations. First, nasopharyngeal RT-PCR was not performed for all participants at baseline, day 7, and 14 because of the limited facilities, so the rate of performed tests was not the same among the two groups. Also, some discharged patients before days 7 and 14 who were asked to refer for repeating the test did not accompany. According to these two issues and the low sensitivity of RT-PCR to detect positive cases ( 34 ), the measured P values of these variables are not reliable. Second, the lack of placebo which was due to the different regimen schedules of the two study arms (ATZ/RTV, one tablet, once daily versus LPV/RTV two tablets, twice daily), and particularly different color and appearance of the two medications. These limitations inevitably changed the blinding manner in which the patient and physician could not be blind to the type of intervention; instead, the investigator and data analyzer were blind. Third, we couldn’t manage to follow the participants after discharge or assign a release sheet for evaluating the time for complete symptom relief and any late complications, even death. It is highly suggested to design a study with long-term follow-up to compare the impact of these regimens on subsequent mortality and the disease recurrence rate. Finally, it is recommended to conduct a trial with a larger sample size for comparing the efficacy of ATZ and LPV in reducing the rate of mortality, ICU-admission, and intubation in moderate COVID-19 that none were significant over the two groups in the present trial.

**Table 1:** Baseline demographic data

***P*** **-value**	**Lopinavir/Ritonavir (n = 62)**	**Atazanavir/Ritonavir (n = 62)**	**Total (n = 124)**	
0.71	36 (58.1)	33 (53.2)	69 (55.6)	Male gender, n (%)
0.38	50.9 ± 12.4	48.9 ± 12.8	49.9 ± 12.6	Age (years), mean ± SD
0.83	7 (4-7)	7 (3.7-7)	7 (4-7)	Time from symptom onset (days), median (IQR_25-75_)
0.08	20 (18-21)	19 (18-20)	20 (18-20)	Respiratory rate (/min), median (IQR_25-75_)
0.90	37.3 (36.8-37.8)	37.2 (36.7-37.8)	37.2 (36.8-37.8)	Temperature (˚C), median (IQR_25-75_)
0.59	29 ± 4.6	29.5 ± 5.3	29.3 ± 4.9	BMI (kg/m^2^), mean ± SD
0.98	85 (80-93)	88.2 (77.9-93.3)	86.6 (79.5-93.3)	MAP (mmHg), median (IQR_25-75_)
0.35	35 (30-45)	40 (30-45)	37.5 (30-45)	Lung CT involvement (%), median (IQR_25-75_)
**Co-morbidities, n (%)**				
0.15	21 (33.9)	13 (21)	34 (27.4)	Diabetes
0.23	14 (22.6)	8 (12.9)	22 (17.7)	IHD
≥0.99	10 (16.1)	11 (17.7)	21 (16.9)	HTN
≥0.99	1 (1.6)	0 (0)	1 (0.8)	COPD
0.49	0 (0)	2 (3.2)	2 (1.6)	Asthma
≥0.99	2 (3.2)	1 (1.6)	3 (2.4)	Hypothyroidism
**Symptoms, n (%)**				
0.69	17 (27.4)	20 (32)	37 (29.8)	Nausea and Vomiting
0.11	6 (9.7)	1 (1.6)	7 (5.6)	Abdominal pain
0.83	17 (25.7)	15 (24.2)	32 (25.8)	Diarrhea
≥0.99	8 (12.9)	7 (11.3)	15 (12.1)	Loss of appetite
**PCR day 1, n (%)**				
0.03	47 (88.7)	30 (69.8)	77 (80.2)	Positive
	6 (11.3)	13 (30.2)	19 (19.8)	Negative
	9 (14.5)	19 (30.6)	28 (22.6)	Not done
**Laboratory findings at baseline, median (IQR**_25-75_ **)**				
0.37	93 (91-94)	93 (92-94)	93 (92-94)	Arterial O_2_ saturation (%)
0.35	12.2 (10.7-13.7)	12.2 (11.6-13.5)	12.2 (11.3-13.7)	Hemoglobin (g/dL)
0.14	167 (140-205.2)	153.5 (126.7-208.7)	159.5 (136.2-206.7)	Platelet (×10^9^/L)
0.44	5.2 (4.2-6.6)	5.2 (4.3-7)	5.2 (4.2-6.9)	WBC (×10^9^/L)
0.98	69.7 (62.9-77.5)	71 (62.6-75.2)	70.5 (63-76)	PMN (%)
0.37	1.2 (0.8-1.4)	1.2 (0.9-1.6)	1.2 (0.9-1.6)	Lymphocyte (×10^9^/L)
0.19	13.2 (10.4-17.6)	12.5 (9.2-16.2)	13 (10-17)	BUN (mg/dL)
0.97	0.9 (0.7-1.1)	0.9 (0.8-1)	0.9 (0.8-1.1)	Cr (mg/dL)
0.02	138 (136-140)	139 (137-141.5)	138 (136-141)	Na (mEq/L)
0.15	4.2 (3.9-4.6)	4.1 (3.9-4.4)	4.1 (3.9-4.5)	K (mEq/L)
0.30	128 (95-239)	112.5 (97-155)	114 (96-182)	Blood sugar (mg/dL)
0.41	30 (22-46)	35 (24-50)	32 (22-49)	AST (U/L)
0.14	33 (21-55)	38 (27-59)	37 (25-56)	ALT (U/L)
0.48	161 (133-218)	160 (130-194)	160 (132-201)	ALP (U/L)
0.32	0.6 (0.5-0.7)	0.6 (0.5-0.7)	0.6 (0.5-0.7)	Bilirubin Total (mg/dL)
0.42	0.3 (0.2-0.4)	0.3 (0.2-0.4)	0.3 (0.2-0.4)	Bilirubin Direct (mg/dL)
0.18	670 (566-787)	630 (489-748)	647 (530-765)	LDH (U/L)
0.85	3 (2-3)	3 (2-3)	3 (2-3)	CRP (/+)
0.75	43 (30-69)	45 (30-66)	44 (30-67)	ESR (mm/h)
0.25	1.2 (1.1-1.3)	1.2 (1.1-1.3)	1.2 (1.1-1.3)	INR
0.70	7.40 (7.38-7.43)	7.41 (7.39-7.43)	7.41 (7.38-7.43)	pH
0.55	26 (24.3-27.9)	26.2 (24.8-28)	26 (24.4-27.9)	HCO_3_ (mEq/L)

BMI: body mass index; MAP: mean arterial pressure; IHD: ischemic heart disease; HTN: hypertension; COPD: chronic obstructive pulmonary disease; PCR: polymerase chain reaction; WBC: white blood cell; PMN: polymorphonuclear; BUN: blood urea nitrogen; Cr: creatinine; AST: aspartate aminotransferase; ALT: alanine transaminase; ALP: alkaline phosphatase; LDH: lactate dehydrogenase; CRP: C-reactive protein; ESR: erythrocyte sedimentation rate; INR: international normalized ratio.

**Table 2:** Primary and secondary outcomes

	**Total (n = 124)**	**Lopinavir/Ritonavir (n = 62)**	**Atazanavir/Ritonavir (n = 62)**	***P*** **-value**
**Primary outcomes**				
Discharge during 10 days, n (%)	96 (72.7)	41 (66.1)	51 (82.3)	0.06
Number of days taking study medication, median (IQR_25-75_)	7 (5-9)	7 (6-9)	6 (5-8)	0.01
**Final outcome**				
Discharge, n (%)	92 (74.2)	41 (66.1)	51 (82.3)	0.03
Death, n (%)	2 (1.6)	2 (3.2)	0 (0)	
Withdrawal of consent, n (%)	2 (1.6)	0 (0)	2 (3.2)	
Release sheet, n (%)	28 (22.6)	19 (30.6)	9 (14.5)	
Final respiratory rate (/min), median (IQR_25-75_)	19 (18-20)	20 (18-22)	18 (18-20)	0.02
Final Arterial O_2_ saturation (%), median (IQR_25-75_)	96 (94-97)	96 (91-97)	97 (95-98)	0.04
**Secondary outcomes**				
Discharge during 14 days, n (%)	102 (82.3)	47 (75.8)	55 (88.7)	0.09
ICU Admission, n (%)	6 (4.8)	3 (4.8)	3 (4.8)	≥0.99
Intubation, n (%)	3 (2.4)	2 (3.2)	1 (1.6)	≥0.99
Mortality Rate, n (%)	2 (1.6)	2 (3)	0 (0)	0.49
Number of days stay in ICU, (max-min)	5	4	5	0.98
Number of days intubated, (max-min)	4	4	3	0.57
**Laboratory tests on day 3, median (IQR**_25-75_ **)**				
CRP	3 (1-3)	3 (1.2-3)	3 (1-3)	0.83
WBC (×10^9^/L)	6 (4.8-8.2)	5.9 (4.7-7.7)	6.2 (4.8-8.3	0.62
PMN (%)	75.4 (64.2-83.1)	75 (62.3-81.4)	75.9 (66.3-84.2)	0.39
Lymphocyte (×10^9^/L)	1.2 (0.9-1.5)	1.2 (0.9-1.5)	1.1 (0.9-1.4)	0.30
Bilirubin total	0.7 (0.6-1.2)	1.1 (0.7-1.7)	0.6 (0.6-0.7)	≤0.001
Bilirubin direct	0.4 (0.3-0.7)	0.6 (0.4-0.9)	0.3 (0.3-0.4)	≤0.001
**Laboratory tests on day 7, median (IQR**_25-75_ **)**				
CRP	1 (0-3)	1 (0-3)	2 (0-3)	0.69
WBC (×10^9^/L)	8.5 (5.2-11.2)	8.5 (4.4-11.1)	8.4 (5.5-13.8)	0.47
PMN (%)	78.5 (69-85.8)	74.3 (68.4-90.5)	79.7 (69-87.7)	0.84
Lymphocyte (×10^9^/L)	1 (0.7-1.3)	0.9 (0.8-1.4)	1 (0.7-1.3)	≥0.99
Bilirubin total	0.8 (0.6-1.2)	1.3 (0.9-1.7)	0.7 (0.6-0.7)	≤0.001
Bilirubin direct	0.4 (0.3-0.7)	0.7 (0.4-0.9)	0.3 (0.3-0.4)	≤0.001
**PCR day 7, n (%)**				
Positive	13 (16.9)	8 (17.2)	5 (16.7)	≥0.99
Negative	27 (35.1)	17 (36.2)	10 (33.3)	
Not done	37 (48)	22 (46.8)	15 (50)	
**PCR day 14, n (%)**				
Positive	3 (23.1)	1 (12.5)	2 (40)	0.50
Negative	7 (53.8)	5 (62.5)	2 (40)	
Not done	3 (23.1)	2 (25)	1 (20)	

CRP: C-reactive protein; WBC: white blood cell; PMN: polymorphonuclear; PCR: polymerase chain reaction.

**Table 3:** Adverse events and other COVID-19 related drugs

	**Total (n = 124)**	**Lopinavir/Ritonavir (n = 62)**	**Atazanavir/Ritonavir (n = 62)**	***P*** **-value**
**Adverse events, n (%)**				
Hemoptysis	4 (3.2)	3 (4.8)	1 (1.6)	0.61
Icterus	2 (1.6)	0 (0)	2 (3.2)	0.49
Dysentery	2 (1.6)	1 (1.6)	1 (1.6)	≥0.99
Dizziness	2 (1.6)	2 (3.2)	0 (0)	0.49
Fever	2 (1.6)	1 (1.6)	1 (1.6)	≥0.99
Nausea and vomiting	8 (6.5)	8 (12.9)	0 (0)	0.006
Hiccups	2 (1.6)	0 (0)	2 (3.2)	0.49
Chest pain	1 (0.8)	1 (1.6)	0 (0)	≥0.99
**Other COVID-19 related drugs, n (%)**				
Ribavirin	1 (0.8)	1 (1.6)	0 (0)	≥0.99
Remdesivir	17 (13.7)	10 (16.1)	7 (11.3)	0.60
Corticosteroid	53 (42.7)	27 (43.5)	26 (41.9)	≥0.99
Intravenous Immunoglobulin (IVIG)	6 (4)	3 (4.8)	2 (3.2)	≥0.99
Interferon	91 (74)	48 (77.4)	43 (70.5)	0.41
NSAIDs	110 (88.7)	58 (93.5)	52 (83.9)	0.15
Azithromycin	63 (50.8)	34 (54.8)	29 (46.8)	0.47
Ceftriaxone	80 (64.5)	36 (58.1)	44 (71)	0.18
Enoxaparin/Heparin	112 (90.3)	54 (87.1)	58 (93.5)	0.36
Favipiravin	5 (4)	4 (6.5)	1 (1.6)	0.36
Tocilizumab	4 (3.2)	2 (3.2)	2 (3.2)	≥0.99
Sofosbuvir/Daclatasvir	23 (18.7)	15 (24.2)	8 (13.1)	0.16

NSAID: non-steroidal anti-inflammatory drug.

## Conclusion

Altogether, the results of this clinical trial showed no significant difference between ATZ/RTV and LPV/RTV regarding the reduced length of hospital stay and improved clinical outcomes in moderate COVID-19. However, ATZ/RTV exerted a more favorable safety profile in comparison with LPV/RTV, and the trend in some clinical aspects was in favor of ATZ/RTV, although statistically, it was not significant. It is still controversy about the efficacy of ATZ/RTV over LPV/RTV in reducing the rate of ICU admission, intubation, and mortality rate, and further investigation is needed.

## Declarations of interest

None.

## Author contributions

H.A.K: Conceptualization; A.R.D.B, A.A and S.A, Clinical assessment of patients; Z.N: Data collection, drafting, and editing manuscript; M.M: Data collection and drafting manuscript; A.M.S: Data collection; S.M: data analysis; S.Ala and A.H: review and edit the final version of the manuscript. All authors critically revised and approved the final submitted version.
